# Ältere Menschen im ersten Jahr der COVID-19-Pandemie: Gesundheitsrelevante Befunde aus dem Deutschen Alterssurvey (DEAS)

**DOI:** 10.1007/s00103-023-03656-w

**Published:** 2023-01-25

**Authors:** Jenna Wünsche, Svenja M. Spuling, Sonja Nowossadeck, Stefan Stuth

**Affiliations:** Deutsches Zentrum für Altersfragen, Manfred-von-Richthofen-Str. 2, 12101 Berlin, Deutschland

**Keywords:** Corona-Pandemie, Gesundheit, Psychosozial, Ältere Erwachsene, DEAS, Covid-19 pandemic, Health, Psychosocial, Older adults, DEAS

## Abstract

Das vorliegende narrative Review führt Befunde des Deutschen Alterssurveys (DEAS) zur gesundheitlichen Lage von Menschen in der zweiten Lebenshälfte im ersten Pandemiejahr zusammen und beantwortet die Frage, ob Ältere, das heißt Menschen im späten Erwachsenenalter, im Vergleich zu Jüngeren, das heißt Menschen im mittleren Erwachsenenalter, in den Anfängen der COVID-19-Pandemie tatsächlich so vulnerabel waren wie vielfach befürchtet. Dabei werden Erkenntnisse zu den folgenden Gesundheitsindikatoren präsentiert: wahrgenommene Bedrohung durch die Corona-Pandemie, erlebte Altersdiskriminierung, selbstberichtete Veränderungen der körperlichen Aktivität, Einsamkeit und subjektive Gesundheit.

Die Ergebnisse zeigen, dass ein höheres Lebensalter nicht pauschal als Risikofaktor für besonders schwerwiegende indirekte Gesundheitsfolgen durch die Corona-Pandemie erachtet werden sollte. Die meisten älteren Menschen haben sich in den Anfängen der Corona-Pandemie kaum durch die Pandemie bedroht gefühlt und nur selten Diskriminierung aufgrund ihres Alters erlebt. Es kam jedoch bei vielen älteren und jüngeren Menschen zu einem selbstberichteten Rückgang an körperlicher Aktivität und zu einem altersunabhängigen Zuwachs an Einsamkeit. Eine Verschlechterung der subjektiven Gesundheit im Vergleich zum präpandemischen Niveau zeichnete sich dagegen nur bei den Älteren ab. Die Verschlechterung der subjektiven Gesundheit scheint jedoch eher auf das individuelle Älterwerden als auf die pandemische Lage zurückführbar zu sein.

Insgesamt wird also deutlich: Bei älteren Menschen in Privathaushalten lässt sich im Vergleich zu jüngeren keine besonders ungünstige gesundheitliche Lage im ersten Jahr der Corona-Pandemie erkennen.

## Abstract (en)

This narrative review brings together findings from the German Ageing Survey (DEAS) on the health situation of people in the second half of life during the first year of the COVID-19 pandemic. In doing so, this review answers the question of whether older adults (i.e., people in late adulthood) were indeed more vulnerable in the early days of the pandemic than younger adults (i.e., people in middle adulthood). Findings on the following health indicators are presented: perceived threat of the COVID-19 pandemic, perceived age discrimination, self-reported changes in physical activity, loneliness, and self-rated health.

The results show that a higher age should not be considered as a universal risk factor for particularly severe indirect health consequences due to the COVID-19 pandemic. Most older adults did not perceive the COVID-19 pandemic as very threatening and rarely experienced discrimination based on their age. By contrast, many younger and older adults reported to be less physically active and they showed an increase in loneliness that was equally distributed across age groups. Moreover, self-rated health deteriorated compared to pre-pandemic levels—but only among older adults. This deterioration, however, seems to be associated with individual ageing rather than the pandemic situation.

It can be concluded that older people in private households did not show a particularly unfavorable health situation in the first year of the COVID-19 pandemic.

## Hintergrund

Die Verbreitung des Coronavirus SARS-CoV‑2 im Jahr 2020 erforderte weitreichende Infektionsschutzmaßnahmen und hat auf diesem Weg das gesellschaftliche Leben grundlegend verändert. Selbstisolation und Quarantäne, Kontaktbeschränkungen und Abstandsregeln, Einschränkungen in der Berufs- und Gewerbefreiheit, Umstellungen auf Heim- oder Kurzarbeit sowie Kita- und Schulschließungen haben den Alltag vieler Menschen beeinflusst und die persönliche Anpassungsfähigkeit erheblich herausgefordert. Auch die medizinische und pflegerische Versorgung im ambulanten wie stationären Bereich war aufgrund der pandemischen Lage erschwert [1]. Es überrascht daher kaum, dass vielerorts auf die möglichen gesundheitlichen Nebenwirkungen der nichtpharmazeutischen Interventionen zur Pandemie-Eindämmung hingewiesen wurde [2].

Hinsichtlich der direkten und indirekten gesundheitlichen Auswirkungen der Corona-Pandemie standen und stehen ältere Menschen im besonderen Fokus der gesellschaftlichen Aufmerksamkeit. Zum einen zählten ältere Menschen und Personen mit Vorerkrankungen zur Risikogruppe für einen besonders schweren oder sogar tödlichen COVID-19-Erkrankungsverlauf [3]. So gehörten etwa 90 % aller bis Dezember 2020 registrierten COVID-19-Todesfälle in Deutschland zur Gruppe der über 70-Jährigen [4]. Jenseits dieser gravierenden direkten gesundheitlichen Auswirkungen durch das unmittelbare Infektionsgeschehen sind außerdem eine Reihe indirekter gesundheitsrelevanter Folgen der Pandemie zu erwarten, von denen ältere Menschen aufgrund der Zugehörigkeit zur Risikogruppe in besonderem Maße betroffen gewesen sein könnten. So wurden ältere Menschen aufgrund des erhöhten Risikos für einen schweren COVID-19-Erkrankungsverlauf verstärkt dazu aufgefordert, sich an geltende Kontaktbeschränkungen zu halten, sei es durch Verzicht auf Enkelbesuche oder das Vermeiden von Supermarkteinkäufen. Dabei ist es in der öffentlichen Debatte häufig zu pauschalisierenden Aussagen über die „vulnerablen Alten“ gekommen [5], die es zu schützen gelte und die sich selbst in angemessener Weise schützen sollten. Während diese Debatten eher den wohlwollenden Schutz älterer Menschen im Fokus hatten, wurde auf der anderen Seite über Triage-Regelungen basierend auf dem Lebensalter diskutiert. Zudem tauchten in sozialen Medien neue Begriffe auf, wie beispielsweise der auf die höhere Sterblichkeitsrate älterer Menschen anspielende Begriff „Boomer Remover“, der als Synonym für das Coronavirus verwendet wurde [6]. Eine Analyse der Twitter-Meldungen im März 2020 ergab, dass etwa ein Viertel der Tweets zum Thema COVID-19 und Alter einen altersdiskriminierenden Inhalt hatten [7].

Insgesamt haben sowohl die wohlwollende, aber pauschalisierende und bevormundende Ansprache älterer Menschen als auch die feindselige Kommunikation in den sozialen Medien deutliche Diskriminierungstendenzen in den Anfängen der Corona-Pandemie zum Ausdruck gebracht. Neben dem faktisch erhöhten Risiko für einen schweren COVID-19-Erkrankungsverlauf könnten diese Dynamiken dazu beigetragen haben, dass sich ältere Menschen in stärkerem Maße als jüngere Vergleichspersonen durch die Pandemie bedroht und aufgrund ihres Alters diskriminiert gefühlt haben. Außerdem wäre erwartbar, dass sich ältere Menschen aufgrund ihrer Zugehörigkeit zur Risikogruppe und der omnipräsenten Erinnerung an diesen Umstand besonders stark aus dem öffentlichen Leben zurückgezogen und folglich ein höheres Risiko hatten, zu vereinsamen oder körperlich inaktiv zu sein. Auf diesem Weg könnte auch die subjektive Gesundheit von älteren Menschen während der Corona-Pandemie stärker beeinträchtigt worden sein als die von jüngeren.

Dieses Negativszenario wird im vorliegenden Beitrag überprüft, indem Befunde des Deutschen Alterssurveys (DEAS) zur wahrgenommenen Bedrohung durch die Corona-Pandemie, zur erlebten Altersdiskriminierung, zu selbstberichteten Veränderungen der körperlichen Aktivität, zur Einsamkeit und zur subjektiven Gesundheit zusammengetragen werden. Dabei werden die Befunde zu Menschen im späten Erwachsenenalter (fortan als Ältere bezeichnet) mit denen von Menschen im mittleren Alter (fortan als Jüngere bezeichnet) verglichen, um herauszuarbeiten, ob Ältere tatsächlich vulnerabler für ungünstige Entwicklungen in ihrem gesundheitlichen Wohlergehen waren oder ob sich die indirekten Folgen der Corona-Pandemie unabhängig vom Alter entfaltet haben.

Interessanterweise liefern bisherige Auswertungen zu ausgewählten Gesundheitsindikatoren keinen Hinweis auf einen einheitlichen Abwärtstrend der gesundheitlichen Lage in Deutschland im ersten Pandemiejahr, weder für ältere noch für jüngere Personen [8–12]. Damerow und Kolleg:innen [8] haben sich auf Daten der Studie Gesundheit in Deutschland aktuell (GEDA 2019/2020-EHIS) gestützt, um die Entwicklung verschiedener Gesundheitsindikatoren (psychische Gesundheit, Rauchverhalten, Gewichtszunahme, soziale Unterstützung und Inanspruchnahme medizinischer Versorgung) zwischen August 2019 und Januar 2021 nachzuzeichnen. Abgebildet wurde die erste Infektionswelle der Pandemie (März bis Mitte Mai 2020), das Sommerplateau (Mitte Mai bis September 2020) und die zweite Infektionswelle (ab Oktober 2020). Insgesamt deuten die Ergebnisse darauf hin, dass sich die Pandemie auf einzelne gesundheitliche Bereiche ausgewirkt hat, wobei insbesondere eine andauernde Gewichtszunahme und ein Rückgang in der Inanspruchnahme medizinischer Versorgung Anlass zur Besorgnis geben könnten. Im Hinblick auf die psychische Gesundheit wurde im Zuge der ersten Pandemiephase keine Verschlechterung, sondern ein temporärer Rückgang an depressiver Symptomatik festgestellt. Hinsichtlich des Rauchverhaltens sowie der erhaltenen, fehlenden und geleisteten Unterstützung ließen sich dagegen keine systematischen pandemiebedingten Veränderungen feststellen. Ein Vergleich zwischen den Altersgruppen machte außerdem deutlich, dass ältere Menschen keine ungünstigeren Gesundheitstrends zeigten als jüngere Personen. Entgegen vieler Befürchtungen legen die Befunde der GEDA-Studie also nahe, dass ältere Menschen (jenseits der direkten gesundheitlichen Folgen einer COVID-19-Erkrankung) nicht als besonders vulnerable Gruppe für negative indirekte Gesundheitseffekte der Pandemie zu erachten sind. Diese Befunde stehen in Einklang mit den Erkenntnissen anderer Untersuchungen zur Gesundheit und zum Gesundheitsverhalten in Deutschland im ersten Pandemiejahr – sowohl hinsichtlich der Gesamtbevölkerung [9, 10] als auch in Bezug auf die spezifische Gruppe älterer Erwachsener [11, 12].

Im vorliegenden narrativen Review werden die Erkenntnisse zur gesundheitlichen Lage älterer Menschen im ersten Pandemiejahr erweitert und vertieft, indem ein besonderes Augenmerk auf ihre subjektiven Wahrnehmungen (wahrgenommene Bedrohung, Altersdiskriminierung, Einsamkeit, subjektive Gesundheit) und selbstberichteten Aktivitätsveränderungen (Sport und Spazierengehen) gelegt wird. Hierzu wird zunächst die Datenbasis des Deutschen Alterssurveys kurz skizziert und es werden ausgewählte Befunde zur Beantwortung dieser Frage präsentiert: Haben sich die besonders herausfordernden Lebensumstände älterer Menschen im ersten Pandemiejahr auch in den subjektiven Wahrnehmungen und im selbstberichteten Aktivitätsverhalten der älteren Bevölkerung niedergeschlagen oder waren ältere Menschen doch deutlich robuster als vielfach vermutet?

## Der Deutsche Alterssurvey in Zeiten der Corona-Pandemie

Der Deutsche Alterssurvey (DEAS; [13, 14]) ist eine bundesweit repräsentative Befragung von Menschen in der zweiten Lebenshälfte. Seit der ersten Befragung des DEAS im Jahr 1996 fanden 6 weitere Längsschnitterhebungen statt (2002, 2008, 2011, 2014, 2017 sowie 2020/2021), wobei 2002, 2008 sowie 2014 jeweils neue Querschnittsstichproben zum DEAS-Panel hinzugefügt wurden. Die Datenerhebung erfolgt in der Regel mittels persönlicher, computergestützter Interviews und über einen zusätzlichen schriftlichen Fragebogen. Das Design und der lange Beobachtungszeitraum machen den DEAS zu einer geeigneten Datenquelle, um die Lebenssituation älterer Menschen in Krisenzeiten – wie etwa der Corona-Pandemie – abzubilden. Hervorzuheben ist jedoch, dass die DEAS-Daten nur für ältere Menschen repräsentativ sind, die in Privathaushalten leben und gesundheitlich dazu in der Lage sind, an einer Befragung teilzunehmen. Die besondere Situation von Menschen in Pflegeheimen wird demnach nicht abgebildet.

Um auf die Herausforderungen im Zusammenhang mit der Corona-Pandemie zu reagieren, wurde das klassische DEAS-Design im Jahr 2020/2021 erweitert und modifiziert. Es wurde erweitert, indem das DEAS-Panel im Sommer 2020 (08.06.–22.07.2020) zu einer zusätzlichen schriftlichen Kurzbefragung zu den Auswirkungen der Corona-Pandemie eingeladen wurde. An dieser Kurzbefragung nahmen 4823 Personen ab einem Alter von 46 Jahren teil (*M* = 69,9, *SD* = 10,4, *Range* = 46–100). Das DEAS-Design wurde zudem modifiziert, indem im Jahr 2020/2021 wegen der pandemischen Lage auf die Erhebung einer neuen Querschnittstichprobe verzichtet wurde, obwohl dies im Rahmen der 6‑Jahres-Taktung vorgesehen gewesen wäre. Weiterhin fanden die Interviews im Jahr 2020/2021 nicht wie sonst persönlich, sondern telefonisch statt. Insgesamt haben an der DEAS-Erhebung im Jahr 2020/2021 (04.11.2020 bis 01.03.2021) 5402 Personen im Alter ab 46 Jahren teilgenommen (*M* = 68,6, *SD* = 10,4, *Range* = 46–100), von denen 4419 Personen auch den schriftlichen Fragebogen bearbeitet haben.

**Figure 1:**
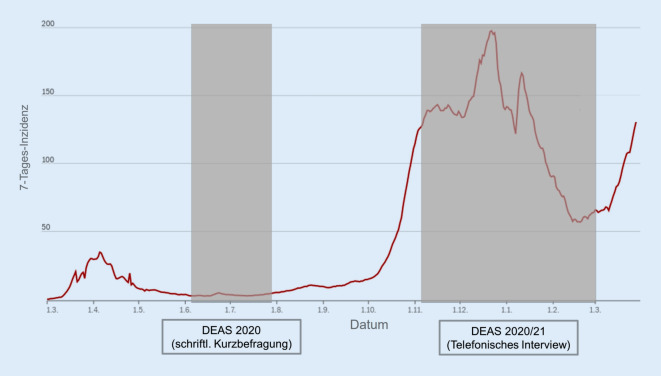

Seit Beginn der Corona-Pandemie fanden also 2 Erhebungen des DEAS statt: eine erste im Sommer 2020 in einer Plateauphase der Corona-Pandemie mit vergleichsweise niedrigen Infektionszahlen und gelockerten Infektionsschutzmaßnahmen und eine zweite im Winter 2020/2021, also inmitten der zweiten Pandemiewelle mit sich dynamisch verändernden Infektionszahlen und zunehmend strengeren Infektionsschutzmaßnahmen. Abb. 1 veranschaulicht die Verortung der stattgefundenen DEAS-Erhebungen im Infektionsgeschehen des ersten Pandemiejahrs.

## DEAS-Befunde zur gesundheitlichen Lage älterer Menschen im ersten Pandemiejahr

### Wahrgenommene Bedrohung durch die Corona-Pandemie

Wettstein und Kolleg:innen [16] haben untersucht, wie stark sich Menschen in der zweiten Lebenshälfte zu Beginn der Corona-Pandemie bedroht gefühlt haben, und sich dabei auf Daten der schriftlichen DEAS-Kurzbefragung im Sommer 2020 gestützt. Von zentralem Interesse war die Frage, ob sich ältere Erwachsene aufgrund ihrer Zugehörigkeit zur Risikogruppe für schwere COVID-19-Erkrankungsverläufe [3] in stärkerem Ausmaß von der Corona-Pandemie bedroht gefühlt haben als jüngere Personen. Erhoben wurde das Bedrohungserleben mit der Frage: „Inwiefern empfinden Sie die Corona-Krise derzeit als Bedrohung für sich selbst?“, die auf einer 10-stufigen Skala von 1 (*überhaupt keine Bedrohung für mich*) bis 10 (*extreme Bedrohung für mich*) beantwortet werden konnte.

**Figure 2:**
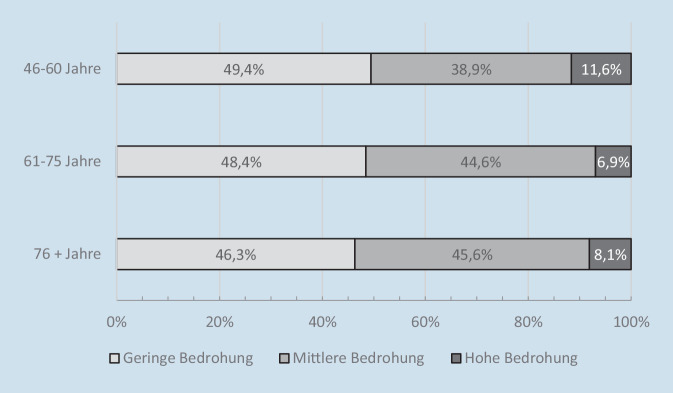

91 % der Menschen in der zweiten Lebenshälfte haben sich nur in geringem (Werte von 1 bis 3) bis mittlerem Maß (Werte von 4 bis 7) durch die Corona-Pandemie bedroht gefühlt. Nicht einmal ein Zehntel der Bevölkerung ab 46 Jahren hat dagegen ein hohes Bedrohungserleben angegeben. Abb. 2 stellt dar, wie sich das Bedrohungserleben über Menschen aus unterschiedlichen Altersgruppen verteilt hat. Erkennbar wird, dass sich Menschen aus der ältesten Altersgruppe der ab 76-Jährigen etwas seltener wenig bedroht gefühlt haben als Befragte aus den beiden jüngeren Altersgruppen. Interessanterweise gaben die ab 76-Jährigen aber auch seltener an, sich in hohem Maße durch die Corona-Pandemie bedroht zu fühlen – zumindest im Vergleich zur Altersgruppe der 46- bis 60-Jährigen. Insgesamt sind die Altersgruppenunterschiede als klein einzuordnen. Ein aufgrund der Zugehörigkeit zur Risikogruppe erhöhtes Bedrohungsempfinden bei älteren Menschen lässt sich demnach aus der Studie von Wettstein und Kolleg:innen [16] nicht ableiten.

### Erlebte Altersdiskriminierung

In einer weiteren Untersuchung haben Wettstein und Nowossadeck [17] Altersdiskriminierungserfahrungen von Menschen in der zweiten Lebenshälfte zu Beginn der Corona-Pandemie beleuchtet. Dabei wurde die Frage aufgeworfen, ob die oftmals negativ gefärbte öffentliche Debatte über ältere Menschen dazu beigetragen hat, dass sich ältere Menschen in dieser Zeit verstärkt aufgrund ihres Alters diskriminiert gefühlt haben. In der schriftlichen DEAS-Kurzbefragung im Sommer 2020 wurden die Teilnehmenden daher gebeten, folgende Frage zu beantworten: „Haben Sie seit Mitte März erlebt, dass Sie wegen Ihres Alters durch andere benachteiligt oder gegenüber anderen Menschen schlechter gestellt wurden?“

**Figure 3:**
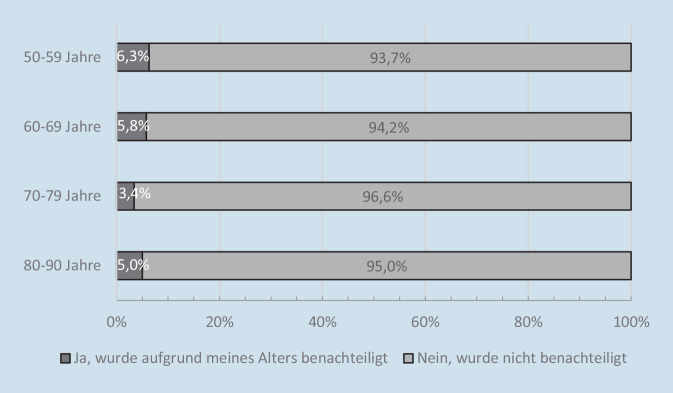

Nur 5,4 % der Menschen in der zweiten Lebenshälfte berichten davon, Altersdiskriminierung seit Beginn der Corona-Pandemie erlebt zu haben. Die große Mehrheit scheint dagegen keiner solchen Diskriminierung ausgesetzt gewesen zu sein. Interessanterweise ergeben sich in der Sommerbefragung 2020 keine statistisch bedeutsamen Zusammenhänge zwischen Alter und Diskriminierungserfahrungen (Abb. 3). Augenscheinliche Unterschiede zwischen der Gruppe der 70- bis 79-Jährigen und den übrigen Altersgruppen sind statistisch nicht signifikant. Das heißt, ältere Menschen waren zu Beginn der Corona-Pandemie zu ähnlichen Anteilen von Diskriminierungserfahrungen aufgrund ihres Alters betroffen wie jüngere Menschen. Die Untersuchung von Wettstein und Nowossadeck [17] legt also nahe, dass trotz der vielfach ungünstigen Darstellung älterer Menschen als schutzbedürftige vulnerable Personengruppe und der damit transportierten negativen Altersstereotypen diskriminierendes Verhalten gegenüber älteren Menschen von diesen sehr selten wahrgenommen wurde. Zu beachten ist allerdings, dass die erlebte Altersdiskriminierung für einen Zeitraum erfasst wurde, in dem strenge Infektionsschutzmaßnahmen galten (März bis Juni/Juli 2020). Aufgrund der Reduktion an sozialen Begegnungen bestanden also auch verhältnismäßig wenige Gelegenheiten für Diskriminierungserfahrungen.

### Selbstberichtete Veränderungen von Sport und Spazierengehen

Die pandemiebedingte Schließung von öffentlichen Sportstätten und die geltenden Kontaktbeschränkungen haben die Ausgangslage für außerhäusliche sportliche Aktivität deutlich verschlechtert und die Ausübung verschiedenster Sportarten unmöglich gemacht. Auch Ängste vor einer Ansteckung mit dem Coronavirus könnten dazu beigetragen haben, dass insbesondere ältere Personen eher auf sportliche Aktivitäten verzichtet haben. Nowossadeck und Kolleg:innen [18] haben aus diesem Grund untersucht, welche Veränderungen in ihrer sportlichen Aktivität Menschen in der zweiten Lebenshälfte seit Beginn der Corona-Pandemie berichteten. Die Befragten konnten in der DEAS-Kurzbefragung im Sommer 2020 angeben, ob sie seit Pandemiebeginn mehr, weniger oder gleich viel Sport getrieben haben („Hat sich Ihre sportliche Aktivität seit Mitte März geändert?“).

**Figure 4:**
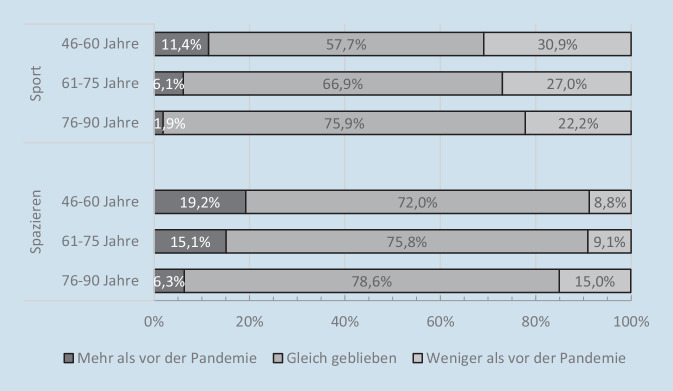

Die Untersuchung von Nowossadeck und Kolleg:innen [18] zeigt, dass mehr als ein Viertel (27,8 %) der Menschen in der zweiten Lebenshälfte angaben, ihre sportlichen Aktivitäten seit Beginn der Corona-Pandemie reduziert zu haben. Immerhin 7,7 % berichteten von einer Steigerung ihrer sportlichen Aktivität. Die große Mehrheit gab jedoch an, dass sie in unverändertem Umfang sportlich aktiv waren (64,5 %). Ein genauerer Blick auf die Veränderungen der sportlichen Aktivität in der ältesten Altersgruppe zeigt (Abb. 4), dass die 76- bis 90-Jährigen im Vergleich zu den beiden jüngeren Altersgruppen die wenigsten Veränderungen berichteten: Im Vergleich zu den unter 76-Jährigen gaben sie am häufigsten an, dass sie im gleichen Umfang sportlich aktiv geblieben sind, und am seltensten, dass sie ihre Aktivität erhöht oder reduziert haben. Zu betonen ist allerdings, dass die älteste Altersgruppe gleichzeitig auch die Gruppe mit dem geringsten Anteil regelmäßig sportlich Aktiver darstellt. Während bei den 46- bis 69-Jährigen im Sommer 2020 62,4 % regelmäßig sportlich aktiv waren, lag dieser Anteil bei den 76- bis 90-Jährigen nur bei 50,7 % (ohne Abbildung).

Doch wie sieht es mit weniger intensiven Formen der körperlichen Aktivität aus, wie beispielsweise dem Spazierengehen? Das Spazierengehen hat in der Corona-Pandemie augenscheinlich an Beliebtheit gewonnen – vermutlich auch, weil es in Phasen mit besonders strengen Infektionsschutzregeln eine der wenigen erlaubten Formen des Beisammenseins im öffentlichen Raum darstellte. Und tatsächlich zeigt die Untersuchung von Nowossadeck und Kolleg:innen [18], dass Spaziergänge bei Menschen in der zweiten Lebenshälfte eher häufiger (15,1 %) als seltener (10,2 %) geworden sind. Insgesamt jedoch hat sich die Häufigkeit des Spazierengehens in den Anfängen der Pandemie weniger stark verändert als die Häufigkeit der sportlichen Aktivität: Der Anteil der Personen, die angaben, seit Pandemiebeginn gleich häufig spazieren zu gehen, lag mit 74,7 % deutlich über dem Anteil der Personen, die angaben, dass ihre sportliche Aktivität unverändert geblieben ist (64,5 %). Die altersdifferenzierte Betrachtung (Abb. 4) zeigt außerdem, dass Personen aus der ältesten Altersgruppe das Spazierengehen am häufigsten reduziert und nur selten ausgeweitet haben.

Insgesamt zeigt die Untersuchung von Nowossadeck und Kolleg:innen [18], dass sportliche Aktivitäten zu Beginn der Corona-Pandemie häufig eingeschränkt wurden. Die Zunahme an Inaktivität war allerdings bei den Ältesten in der Bevölkerung nicht stärker ausgeprägt als in den jüngeren Altersgruppen. Nichtsdestotrotz ist der Anteil der regelmäßig sportlich Aktiven in der ältesten Altersgruppe als besorgniserregend niedrig einzustufen. Hinsichtlich der leichten körperlichen Aktivität, in Form des Spazierengehens, scheint dagegen die ungünstige pandemische Ausgangslage für die Ältesten am ehesten zu einem Rückgang an Aktivität beigetragen zu haben.

### Einsamkeit

Eine zentrale Sorge im Hinblick auf die möglichen indirekten Folgen der Pandemie-Eindämmungsmaßnahmen betraf die Gefährdung des sozialen Wohlbefindens in der Bevölkerung. Gewarnt wurde insbesondere vor einem Anstieg der Einsamkeit, der sich daraus hätte ergeben können, dass die drastisch reduzierten sozialen Kontaktmöglichkeiten nicht genügten, um das persönliche Bedürfnis nach zwischenmenschlicher Nähe und Zugehörigkeit zu erfüllen. Da ältere Menschen aufgrund ihres erhöhten Risikos für einen schweren COVID-19-Erkrankungsverlauf zu besonderer Vorsicht angehalten wurden, wäre es denkbar, dass sich bei ihnen eine besonders große Diskrepanz zwischen den tatsächlichen und den gewünschten sozialen Begegnungen ergeben hat. Huxhold und Tesch-Römer [19] haben auf Grundlage der DEAS-Daten aus den Jahren 2014, 2017 und der Kurzbefragung im Sommer 2020 untersucht, ob sich das Einsamkeitsrisiko zu Beginn der Corona-Pandemie bei Menschen in der zweiten Lebenshälfte erhöht hat und ob sich dieser Trend bei älteren Menschen tatsächlich in besonders starkem Maß abzeichnet. Das Einsamkeitserleben wurde zu allen Erhebungszeitpunkten mittels 6 Items (z. B. „Ich vermisse Geborgenheit und Wärme“) auf einer vierstufigen Skala [20] von 1 (*trifft genau zu*) bis 4 (*trifft gar nicht zu*) erfasst. Als „einsam“ wurden Personen eingeordnet, wenn sie einen Skalenmittelwert von mehr als 2,5 aufwiesen.

**Figure 5:**
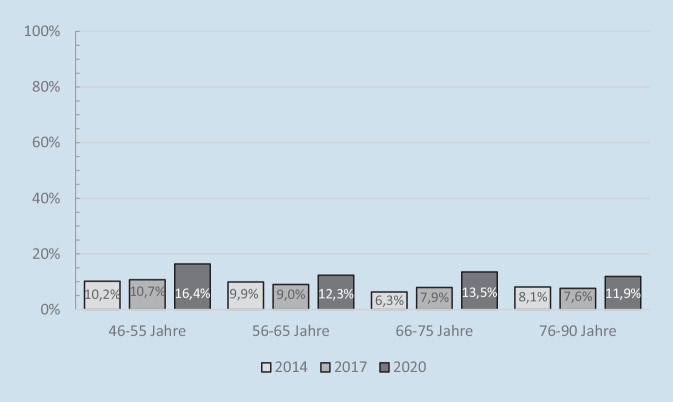

Die Untersuchung von Huxhold und Tesch-Römer [19] zeigt, dass die Einsamkeitsrate bei Menschen in der zweiten Lebenshälfte nach Beginn der Corona-Pandemie deutlich erhöht war: Während das Einsamkeitsrisiko in den Jahren 2014 und 2017 bei etwa 9 % lag, ließ sich im Sommer 2020 ein Einsamkeitsrisiko von 13,7 % erkennen. Die Studie zeigt außerdem, dass keine statistisch bedeutsamen Unterschiede im Einsamkeitszuwachs zwischen den Altersgruppen bestanden (Abb. 5). Daraus lässt sich schließen, dass die Corona-Pandemie in ihren Anfängen tatsächlich das soziale Wohlbefinden erheblich beeinträchtigt hat. Im Gegensatz zur öffentlichen Erwartung betrifft diese ungünstige Pandemiefolge jedoch nicht allein die Ältesten in der Bevölkerung.

### Subjektive Gesundheit

Die vielfältigen direkten und indirekten gesundheitsrelevanten Herausforderungen der Corona-Pandemie könnten letztlich auch auf das Gesundheitsempfinden der Menschen in dieser Zeit Einfluss genommen haben. Aus diesem Grund haben Stuth und Wünsche [21] die subjektive Gesundheit während der Corona-Pandemie untersucht. Dabei wurde auch die Rolle des Lebensalters für Veränderungen in den Gesundheitseinschätzungen in den Blick genommen. Um Aufschluss über die pandemiebedingten Entwicklungen der subjektiven Gesundheit von Menschen in der zweiten Lebenshälfte zu gewinnen, wurden nur Angaben von Personen ausgewertet, die an allen 3 DEAS-Erhebungen in den Jahren 2014, 2017 und im Winter 2020/2021 teilnahmen. Im Gegensatz zu den zuvor beschriebenen Studien bildet diese Untersuchung also nicht die Situation während des Sommerplateaus 2020 ab. Stattdessen wird die Lage während der zweiten Pandemiewelle mit zunehmend strengeren Infektionsschutzmaßnahmen dargestellt. Hervorzuheben ist außerdem, dass nur die Gesundheitseinschätzungen von Personen betrachtet wurden, die sich selbst bis zu diesem Zeitpunkt (noch) nicht mit dem Coronavirus infiziert hatten. Diese Restriktion wurde vorgenommen, um den direkten Gesundheitseinfluss einer Coronaviruserkrankung von den indirekten Gesundheitsfolgen der Infektionsschutzmaßnahmen trennen zu können. Zur Erfassung der subjektiven Gesundheit wurden die DEAS-Teilnehmenden gefragt: „Wie bewerten Sie Ihren derzeitigen Gesundheitszustand?“ Dabei konnten sie ihre Einschätzung auf einer Skala von 1 (*sehr gut*) bis 5 (*sehr schlecht*) abstufen.

**Figure 6:**
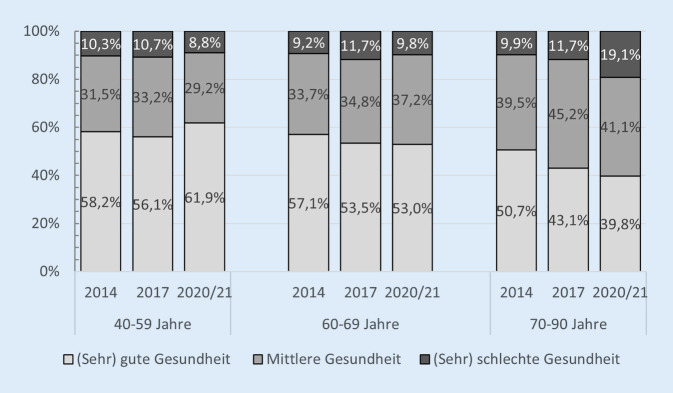

Es zeigte sich, dass sich die subjektive Gesundheit der Menschen in der zweiten Lebenshälfte im Vergleich zwischen 2017 und dem Winter 2020/2021 nicht in statistisch bedeutsamem Ausmaß verändert hat. Im Winter 2020/2021 kam die Mehrheit der Befragten (52,6 %) zu einer guten oder sehr guten Einschätzung ihrer Gesundheit und nur 12,4 % der Menschen bewerteten ihre Gesundheit als schlecht oder sehr schlecht. Ein Vergleich der gesundheitlichen Entwicklung in den verschiedenen Altersgruppen (Abb. 6) ergibt jedoch ein differenzierteres Bild der gesundheitlichen Lage in der zweiten Lebenshälfte. So haben Stuth und Wünsche [21] bei der Altersgruppe der 70- bis 90-Jährigen einen Abwärtstrend der subjektiven Gesundheit zwischen 2017 und dem Winter 2020/2021 festgestellt. Da sich bei der ältesten Altersgruppe allerdings auch schon zwischen 2014 und 2017 ein Verschlechterungstrend zeigte, kann nicht davon ausgegangen werden, dass die weitere Verschlechterung der subjektiven Gesundheit allein auf die Pandemie zurückzuführen ist. Stattdessen könnte der gesundheitliche Abwärtstrend eher durch das Älterwerden zwischen den Befragungszeitpunkten begünstigt worden sein. Bei den unter 70-Jährigen deutet sich hingegen ein günstiger Effekt der Corona-Pandemie auf die subjektive Gesundheit an. So zeigt sich bei den 40- bis 59-Jährigen zwischen 2017 und dem Winter 2020/2021 ein Aufwärtstrend in den Gesundheitseinschätzungen, der zwischen 2014 und 2017 noch nicht erkennbar war. In der mittleren Altersgruppe der 60- bis 69-Jährigen deutet sich wiederum zwischen 2017 und 2020/2021 eine Stabilisierung der subjektiven Gesundheit an, nachdem bei dieser Altersgruppe zwischen 2014 und 2017 ein gesundheitlicher Abwärtstrend erkennbar war. Die Befunde von Stuth und Wünsche [21] geben demnach keinen eindeutigen Grund zur Annahme, dass sich die Gesundheitseinschätzungen der Ältesten (die noch keine COVID-19-Erkrankung hatten) in besonderem Maße unter den Herausforderungen der Corona-Pandemie verschlechtert haben.

## Fazit und Ausblick

Der vorliegende Beitrag hat zentrale DEAS-Befunde zusammengetragen, um die gesundheitliche Lage von Menschen in der zweiten Lebenshälfte im ersten Pandemiejahr zu beleuchten und die Frage zu beantworten, ob ältere im Vergleich zu jüngeren Personen in den Anfängen der Corona-Pandemie tatsächlich so vulnerabel waren wie vielfach befürchtet. Dabei wurden 6 Gesundheitsindikatoren betrachtet, die jeweils einen wertvollen ergänzenden Beitrag zum bisherigen Forschungsstand leisten. Es wurde deutlich, dass sich ältere Menschen in den Anfängen der Corona-Pandemie eher wenig – zumindest aber nicht stärker als jüngere Personen – durch die Pandemie bedroht gefühlt und Diskriminierung aufgrund ihres Alters erlebt haben. Auch hinsichtlich der sportlichen Aktivität und des sozialen Wohlbefindens haben ältere Menschen im Vergleich zu jüngeren keine betonten Verschlechterungen gezeigt. Einbußen in diesen beiden Bereichen wurden dennoch deutlich und äußerten sich in einem selbstberichteten Rückgang an sportlicher Aktivität und einem Zuwachs an Einsamkeit. Einzig für das Spazierengehen und die subjektive Gesundheit zeichnete sich bei älteren Menschen eine ungünstigere Entwicklung ab. Zum einen berichteten die Ältesten vergleichsweise häufiger von weniger Spaziergängen als jüngere Personen und zum anderen zeigten sie einen Rückgang an subjektiver Gesundheit, der bei den jüngeren Altersgruppen nicht zu erkennen war. Der Rückgang an subjektiver Gesundheit bei den Ältesten ist allerdings nicht eindeutig auf die Corona-Pandemie zurückführbar und sollte eher als Verschlechterungstrend eingeordnet werden, der mit dem individuellen Älterwerden einhergegangen ist. Diese Befunde stehen im Einklang mit bisherigen Untersuchungen, die ebenfalls darauf hindeuten, dass die ältere Bevölkerung in Deutschland im Hinblick auf viele gesundheitsrelevante Indikatoren resilient gegenüber den Herausforderungen des ersten Pandemiejahrs gewesen ist [8, 11] und dass beobachtbare ungünstige Entwicklungen – wie beispielsweise im Hinblick auf den Anstieg an Einsamkeit – nicht an das Lebensalter gebunden sind [12].

Auf Grundlage der in diesem Beitrag präsentierten Befunde und der Erkenntnisse aus anderen Studien lässt sich demnach eine zentrale Botschaft ableiten: Die Annahme einer generell erhöhten Vulnerabilität älterer Menschen hinsichtlich der indirekten Gesundheitsfolgen der Corona-Pandemie ist nicht haltbar. Das Lebensalter allein sollte also nicht als Risikomarker für ungünstige indirekte Gesundheitsfolgen der Corona-Pandemie herangezogen werden. Viel mehr deuten die Befunde darauf hin, dass eine indikatorspezifische Identifikation von Risikogruppen notwendig ist. So wäre eine gezielte Ansprache älterer Menschen zur Förderung leichter körperlicher Aktivität (z. B. Spaziergänge) durchaus sinnvoll. Im Hinblick auf Maßnahmen zur Erhöhung des sozialen Wohlbefindens erscheint es dagegen plausibler, ein breiteres Angebot zu schaffen, da Menschen aus verschiedenen Altersgruppen gleichermaßen von der Erhöhung des Einsamkeitsrisikos im ersten Pandemiejahr betroffen waren.

Nichtsdestotrotz sollte hervorgehoben werden, dass die hier präsentierten Befunde nicht repräsentativ für das gesundheitliche Wohlergehen von älteren Menschen in Pflegeeinrichtungen oder mit schweren gesundheitlichen Beeinträchtigungen sind, da diese Personen nur in einem äußerst geringen Umfang in der DEAS-Stichprobe vertreten sind [14]. Weiterhin ist zu beachten, dass es sich bei den Befunden lediglich um Momentaufnahmen (vom Sommer 2020 bzw. Winter 2020/2021) der gesundheitlichen Lage älterer Menschen innerhalb des ersten Pandemiejahrs handelt, das hinsichtlich Infektionszahlen und Eindämmungsmaßnahmen äußerst dynamisch verlaufen ist. Beide Beobachtungszeitpunkte haben jedoch gemein, dass bestehende Infektionsschutzmaßnahmen vor allem auf Kontaktbeschränkungen abzielten. Selbsttests und Impfungen standen dagegen bis zum Winter 2020/2021 noch nicht flächendeckend zur Verfügung. Weitere Beobachtungszeitpunkte sind daher notwendig, um zu untersuchen, ob sich im weiteren Verlauf der Corona-Pandemie – und den sich wandelnden Infektionsschutzmaßnahmen – Erholungs‑, Chronifizierungs- oder sogar Verschärfungseffekte ergeben haben. Die aktuellste DEAS-Erhebung vom Winter 2022/2023 wird hierzu Aufschlüsse geben können.
